# Supplementary figures and images for: Overexpression of Escherichia coli yaiX Confers Multidrug Resistance and Enhances Virulence in the Silkworm Infection Model
Source: Microbiol Immunol. 2026 Feb 25;70(4):193–205. doi: 10.1111/1348-0421.70049 (PMC13058169; doi:10.1111/1348-0421.70049)

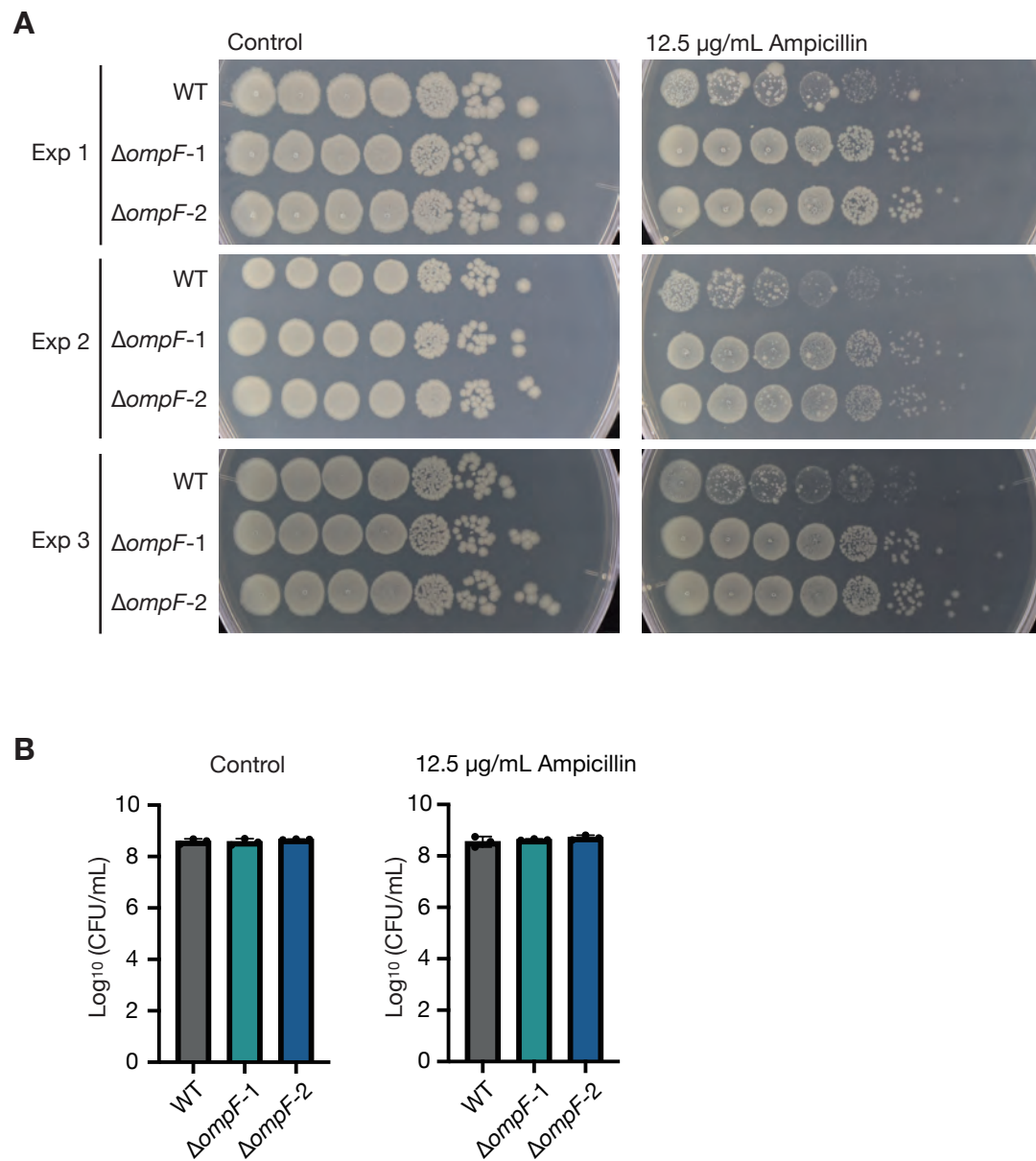

**Figure S1**

Supplement: Supplementary file 1 — Figure S1: The ompF deletion mutant forms larger colonies than the wild‐type strain in the presence of ampicillin [file MIM-70-193-s002.pdf]
